# High risk of essential hypertension in males with intron 4 VNTR polymorphism of *eNOS* gene

**DOI:** 10.4103/0971-6866.55215

**Published:** 2009

**Authors:** Sushma Patkar, B. H. Charita, C. Ramesh, T Padma

**Affiliations:** Department of Genetics, Osmania University, Hyderabad - 500 007, India; 1 Gandhi Medical College and Hospital, Hyderabad - 500 007, India

**Keywords:** Essential hypertension, endothelial NOS gene, polymorphism

## Abstract

In this study 250 patients with essential hypertension were investigated in comparison to 218 normotensives for association with epidemiological parameters. Of these DNA samples from 176 patients and 168 controls were analyzed for intron 4 27bp repeat polymorphism of *eNOS* gene. The study revealed significantly high risk of essential hypertension for individuals who were obese, with a positive family history and with non-vegetarian food habits. Though the intron 4b/a polymorphism of *eNOS* gene did not reveal any association with essential hypertension in general, males with a/a genotype of the polymorphism did show significantly high risk for developing hypertension.

## Introduction

Hypertension is a multi-factor disease involving interaction of both environment and genetic components. It is a major risk factor for Coronary Artery Disease (CAD) which is associated with high mortality rate. Clinical and experimental studies have suggested involvement of several genes in the causation of hypertension. Alteration in Nitric Oxide (NO) metabolism is considered a major contributing factor. NO, a powerful endogenous vasodilator, is synthesized by endothelial Nitric Oxide Synthase gene (*eNOS*)[1,2] and inhibits the adhesion, and recruitment of platelets,[3,4] vascular smooth muscle cell migration and its growth. It also limits the oxidation of atherogenic low density lipoproteins.

The constitutive endothelial NOS (*eNOS*) expressed in endothelium is encoded by a gene on chromosome 7q35-36 position. It comprises of 26 exons that spans 21 kilo bases encoding an mRNA of 4052 nucleotides.[5,6] Several polymorphisms of *eNOS* gene are found to be associated with increased risk for CVD. Of these 894 G greater than T variant in exon 7 is reported to be associated with CAD[7,8] while 786 T greater than C polymorphism has been associated with Hypertension;[9] and with coronary spasm.[10] It is considered a risk factor for CAD in Caucasians.[11]

A 27 bp VNTR located in intron 4 of *eNOS* gene was proven to be of equal interest. Wang *et al*.[12] reported a significant association of this intron with CAD. The study identified two alleles in intron 4, the larger allele with five tandem repeat units of 27 bps with first three having A and the last two G at 19^th^ position of the repeat unit [GAAGTCTAGACCTGCTGC(A/G)GGGGTGAG] and the smaller allele with only four repeats, in which the first two repeats had A and the last two had G at the 19^th^ position. The authors have designated these alleles as *eNOS* 4a for shorter allele with four repeats and *eNOS* 4b for larger allele with five repeats. The study also showed that homozygosity for allele 4a had higher risk for CAD among smokers.

Other studies on *eNOS* intron 4 polymorphism showed positive association with renal disease[13,14] essential hypertension among Japanese[15] and stroke among Chinese.[16] However, studies from Taiwan[17] did not reveal any association with premature CAD. The discrepancy in these studies on the association of *eNOS* intron 4b/a VNTR polymorphism with Essential Hypertension may be related to ethnic diversity. Hence we investigated this polymorphism in Indian patients with Essential Hypertension to find the extent of risk, caused by this gene, if any.

## Materials and Methods

### Study subjects

The study population included 250 individuals with essential hypertension diagnosed according to World Health Organization (WHO) criteria. Subjects included in the study comprised of those who were diagnosed as primary hypertensives by physicians (based on clinical and other investigations) and those who were already on antihypertensive drugs at the time of study. The patients were recruited at Gandhi Medical College and Hospital, Hyderabad, India. It is well equipped with diagnostic facilities. Patients of all socio-economic groups visit this hospital. Subjects diagnosed with secondary hypertension arising due to CAD, renal failure and other associated conditions were excluded from the study. The data generated was analyzed in comparison to that found in 218 normotensive healthy subjects that formed a control group.

Detailed information relating to the age, sex, BMI, Family history, consanguinity, diet and habits like alcohol consumption and smoking were collected from both patients and controls. Family information in terms of a four generation pedigrees was also obtained. Of the cases and controls registered for the study, 5 ml of venous blood was collected in EDTA vaccutainers from 176 cases and 168 controls. Informed consent was obtained from each individual for their participation in the study which was approved by the Ethical Committee of our institution.

### Analysis of VNTR Polymorphism of *eNOS*

Genomic DNA was extracted from peripheral blood leukocytes by non-enzymatic method.[18] Genotypes for *eNOS* polymorphism were determined by polyacrylamide gel electrophoresis after PCR amplification (Biometra, Germany) of the target region. The primers used for amplification were forward 5'- AGGCCCTATGGTAGTGCCTT

T -3' and the reverse 5'- TCTCTTAGTGCTGTGGTC AC -3' that flank the region of the 27 bp repeat in intron 4 of *eNOS* gene.[13] Each reaction mixture was heated to initial denautrarion of 94°C for 4minutes with 35 cycles of denaturation at 94°C for 1minute, annealing at 56°C for one minute, extension at 72°C for two minutes and a final extension at 74°C for seven minutes.

**Figure 1:**
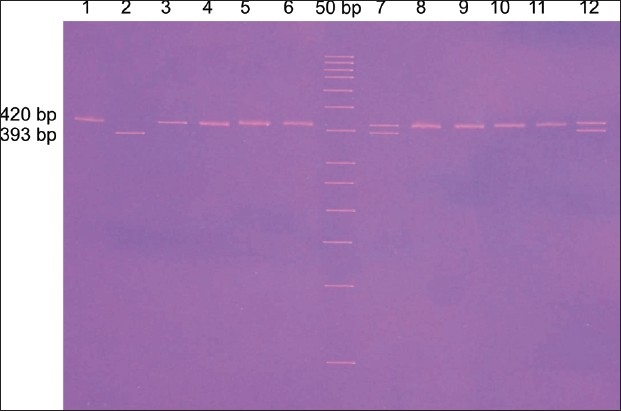
VNTR gene polymorphism (393bp for 4a/a homozygous lane 2, 420bp for 4 b/b homozygous Lanes 1,3,4,5,6,8,9,10,11and 4 b/a heterozygous are lanes 7 & 12)

The PCR products were run on eight per cent PAGE gels (Biotech, Indigenous), and the fragments separated were visualized by ethidium bromide staining under UV trans-illumination. PCR analysis of genomic DNA generated fragments of 393 bps corresponding to 4a/a homozygotes, 420 bp to 4b/b homozygotes and 393 and 420 bp 4b/a heterozygote [Figure 1].

The data was analyzed using descriptive statistics for epidemiological parameters and test of significance and odds ratio estimation to evaluate the risk of VNTR genotypes at *eNOS* locus in terms of association with hypertension.

## Results

Table 1 shows the base line features found in the patient and control groups. The mean blood pressure recorded in hypertensive patients was 152.83 plus/minus 23.28 for SBP and 95.55 plus/minus 14.12 for DBP. The males and females occurred with equal incidence in the present hypertensive patients with the mean age group of 52.73 plus/minus 11.52. The frequency of case with positive family history was significantly higher in hypertensives (51.2%) as compared to controls [48.2%; χ^2^ =8.105, *P* = 0.004] suggesting the presence of strong genetic component underlying hypertension. The frequency of obese individuals was significantly higher among patients (13.2%) as compared to controls [2.75%; χ^2^ =16.64, *P* = 0.00005]. Similarly, the frequency of non-vegetarians was found to be higher in the patient group (85.6%) as in comparison to controls (77.06%; χ^2^ =5.657, *P* = 0.017). There was no significant deviation seen among smokers and alcoholics between patient group and control groups.

**Table 1:** Distribution of epidemiological parameters in hypertensive and normotensive groups

	Hypertensives		Controls		χ^2^
	n	%	n	%	
Total	250		218		
Male	125	50.0	131	60.09	4.78*
Female	125	50.0	87	39.90	*P* = 0.028
Obese	33	13.2	58	2.75	16.64*
Non-obese	217	86.8	160	97.25	*P* = 0.00004
Smokers	55	22.0	82	26.60	1.34
Non-smokers	195	78.0	136	73.39	*P* = 0245
Alcoholic	95	38.0	83	37.61	0.007
Non-alcoholic	155	62.0	135	62.38	*P* = 0.993
Familial	128	51.2	50	38.07	8.10*
Non-familial	122	48.2	168	61.92	*P* = 0.004
Veg	36	14.4	6	22.93	5.65*
Non-veg	214	85.6	212	77.06	*P* = 0.017
SBP	152.83 ± 23.28			
DBP	95.55 ± 14.12			

The analysis of polymorphism at *eNOS* intron-4 showed an incidence of 65.9% of b/b, 28.4% of b/a and 5.6% of a/a genotypes in cases and 69% of b/b, 27.9% of b/a, 2.9% of a/a among controls [Table 2]. The gene and genotypic frequencies in hypertensives and controls were in agreement with Hardy-Weinberg Equilibrium. The distribution of *eNOS* genotypes when tested didn't show significant deviation between patients and controls [χ^2^ = 1.57 with 2 df, *P* = 0.455]. However significant deviation was observed between male patients and male controls where b/a heterozygotes (31.4%) and a/a homozygotes (9.3%) were higher in the patient group as compared to controls [b/a 26.0% and a/a 2.0%; χ^2^ = 6.18, *P* = 0.045]. A significant deviation in distribution of genotypes was also observed between males and females within hypertensives group where in the frequency of b/b genotypes was higher in females (72.2%) as compared to males [59.3%; χ^2^ = 5.52, *P* = 0.06]. To compute the odd's ratios the genotypic frequencies were pooled in different combinations to estimate the risk for the condition. When distribution of b/b genotype was compared with pooled genotypic combinations of b/a and a/a, a significant deviation was seen in males [χ^2^ =3.32, *P* = 0.068, OR = 0.566, with 0.307-1.066 CI at 10% level]. In compliance to this when a/a genotype was compared to pooled b/b and b/a genotypes, a significant result was obtained [χ^2^ =4.846, *P* = 0.027, OR = 5.025, with 1.307-24.34 CI]. Further when the frequencies of 4b/a alleles were tested, significant OR was obtained in males supporting high risk of hypertension for males carrying allele 'a' [χ^2^ =2.93, *P* = 0.086, OR = 0.529,with 0.254-1.1035 CI].

**Table 2:** Distribution of *eNOS* intron 4 b/a genotypes with reference to epidemiological parameters in hypertensives and normotensives

	Hypertensives							Controls						
	b/b		b/a		a/a		Total N	b/b		b/a		a/a		Total N
	n	%	n	%	n	%		n	%	n	%	n	%	
Total	116	65.9	50	28.4	10	5.6	176	116	69.0	47	27.9	5	2.9	168
Male	51	59.3	27	31.4	8	9.3	86	72	72	26	26	2	2	100*
Female	65	72.2	23	25.5	2	2.2	90	44	64.7	21	30.8	3	4.4	68
Obese	17	65.4	8	30.8	1	3.8	26	5	83.3	1	16.7	0	0	6
Non-obese	99	66.0	42	28.0	9	6.0	150	111	68.5	46	28.4	5	3.1	162
Smokers	21	55.2	15	39.4	2	5.2	38	27	69.2	11	28.2	1	2.5	39
Non-smokers	96	69.02	35	25.1	8	5.7	139	89	68.9	36	27.9	4	3.1	129
Alcoholic	40	62.5	20	31.3	4	6.3	64	40	68.9	17	29.3	1	1.7	58
Non-alcoholic	76	67.8	30	26.7	6	5.3	112	76	69.1	30	27.2	4	3.6	110
Familial	60	62.5	31	32.2	5	5.2	96	49	70.0	21	30.0	0	0	70
Non-familial	55	68.7	20	25	5	6.3	80	67	68.3	26	26.5	5	5.1	98

χ^2^ =6.18,

* *P* = 0.045

## Discussion

It was demonstrated that 27bp repeat in the *eNOS* gene could bind nuclear proteins as an enhancer/repressor to promote/suppress the transcription efficiency.[19] Functional significance of this polymorphism was also identified in cases with endothelial dysfunction.[20] In the present study the frequency of 4a allele was found to be approximately 0.16, which is little higher than that found in other populations viz., Iranian (0.1), Japanese (0.1 to 0.13) and Turkish (0.14) but is slightly lower than in Australian (0.17) and African Americans (0.26).[21] The differences in the ethnic origin and sample sizes studied might have caused the differences in the distribution of *eNOS* intron 4a polymorphism studied in these populations. With reference to diseases Wang *et al*.[12] showed a significant association of *eNOS* polymorphism with CAD in smokers but not with hypertension. Uwabo *et al*.[15] have shown a significant association of hypertension with a/a homozygosity among the Japanese. They also suggested that this VNTR polymorphism may be in linkage disequilibrium with other genes related to essential hypertension. The report of Ichihara *et al*,[22] and Salimi *et al*.,[21] have shown a positive association of *eNOS* 4b/a polymorphism with CAD in Japanese and Iranian populations. Absence of such association was reported in German and Taiwanese populations.[23,17]

Though this study did not reveal significant association of *eNOS* intron4 polymorphism with essential hypertension in general, males with carriers for allele 'a' did show a significant risk for the condition which was five times higher compared to other genotypes. Further, individuals who were obese, with positive family history, and non-vegetarian food habits were also at risk as evidenced by the high occurrence of hypertensives in these groups. Contribution of these risk factors has to be evaluated with large samples and cohorts of confounding factors.
